# Vital Signs: Human Immunodeficiency Virus Testing and Diagnosis Delays — United States

**DOI:** 10.15585/mmwr.mm6647e1

**Published:** 2017-12-01

**Authors:** Andre F. Dailey, Brooke E. Hoots, H. Irene Hall, Ruiguang Song, Demorah Hayes, Paul Fulton, Joseph Prejean, Angela L. Hernandez, Linda J. Koenig, Linda A. Valleroy

**Affiliations:** 1 Division of HIV/AIDS Prevention, National Center for HIV/AIDS, Viral Hepatitis, STD, and TB Prevention, CDC.

## Abstract

**Background:**

Persons unaware of their human immunodeficiency virus (HIV) infection account for approximately 40% of ongoing transmissions in the United States. Persons are unaware of their infection because of delayed HIV diagnoses that represent substantial missed opportunities to improve health outcomes and prevent HIV transmission.

**Methods:**

Data from CDC’s National HIV Surveillance System were used to estimate, among persons with HIV infection diagnosed in 2015, the median interval (and range) from infection to diagnosis (diagnosis delay), based on the first CD4 test after HIV diagnosis and a CD4 depletion model indicating disease progression and, among persons living with HIV in 2015, the percentage with undiagnosed infection. Data from CDC’s National HIV Behavioral Surveillance were analyzed to determine the percentage of persons at increased risk for HIV infection who had tested in the past 12 months and who had missed opportunities for testing.

**Results:**

An estimated 15% of persons living with HIV in 2015 were unaware of their infection. Among the 39,720 persons with HIV infection diagnosed in 2015, the estimated median diagnosis delay was 3.0 years (interquartile range = 0.7–7.8 years); diagnosis delay varied by race/ethnicity (from 2.2 years among whites to 4.2 years among Asians) and transmission category (from 2.0 years among females who inject drugs to 4.9 years among heterosexual males). Among persons interviewed through National HIV Behavioral Surveillance, 71% of men who have sex with men, 58% of persons who inject drugs, and 41% of heterosexual persons at increased risk for HIV infection reported testing in the past 12 months. In each risk group, at least two thirds of persons who did not have an HIV test had seen a health care provider in the past year.

**Conclusions:**

Delayed HIV diagnoses continue to be substantial for some population groups and prevent early entry to care to improve health outcomes and reduce HIV transmission to others.

**Implications for Public Health Practice:**

Health care providers and others providing HIV testing can reduce HIV-related adverse health outcomes and risk for HIV transmission by implementing routine and targeted HIV testing to decrease diagnosis delays.

## Introduction

Persons unaware of their human immunodeficiency virus (HIV) infection are estimated to account for approximately 40% of ongoing transmissions in the United States (*1*). As a result of increased testing, the percentage of persons living with HIV who are aware of their infection has steadily increased; at the end of 2014, an estimated 85% of persons living with HIV were aware of their infection, approaching the national goal of 90% by 2020 (*2*). Persons aware of their HIV infection reduce their transmission risk behaviors and can enter HIV care and take antiretroviral treatment to achieve viral suppression (a viral load result of <200 copies/mL, or undetectable levels) (*3*). Viral suppression not only preserves immune function, decreasing a person’s risk for morbidity and mortality, but also profoundly reduces risk for sexual transmission to others (*4*–*6*). Early detection of HIV infection maximizes these benefits.

CDC recommends routine testing for HIV infection for persons aged 13–64 years in health care settings and testing at least annually for persons at high risk for HIV infection (*7*). Yet, according to National HIV Behavioral Surveillance (NHBS), one third of gay, bisexual, and other men who have sex with men (MSM) have not been tested in the past year, with even lower percentages of recent testing reported among other population segments at high risk for HIV infection.

## Methods

Data reported to CDC’s National HIV Surveillance System from 50 states and the District of Columbia through June 2017 were used to estimate the total number of persons living with HIV infection (diagnosed and undiagnosed infection, or prevalence) at year-end 2015 and the median number of years and interquartile range between infection and diagnosis (diagnosis delay) of persons with HIV diagnosed in 2015 (*8*,*9*). The first CD4 test after HIV diagnosis and a CD4 depletion model indicating disease progression were used to estimate year of infection and the distribution of time from HIV infection to diagnosis among persons with diagnosed infection (*9*). The distribution of diagnosis delay was used to estimate the annual number of HIV infections, which includes persons with diagnosed infection and persons with undiagnosed infection. HIV prevalence (persons with diagnosed or undiagnosed HIV infection) was estimated by subtracting reported cumulative deaths among persons with HIV infection from cumulative HIV infections.

The number of persons with undiagnosed HIV infection was estimated by subtracting the number of reported cumulative diagnoses from the number of estimated cumulative infections. The percentage of undiagnosed infections was determined by dividing the number of undiagnosed infections by the total HIV prevalence.

Data from NHBS were used to determine the percentage of persons at increased risk for infection who were tested in the past 12 months and the percentage who missed opportunities for testing.* NHBS monitors HIV-associated behaviors and HIV prevalence in cities^†^ with high acquired immunodeficiency syndrome (AIDS) prevalence among three populations with HIV risk behaviors: MSM, persons who inject drugs, and heterosexual persons at increased risk for HIV infection.

Cross-sectional data reported in this analysis are from MSM, persons who inject drugs, and heterosexual persons at increased risk for HIV infection recruited for face-to-face interviews and HIV testing through venue-based sampling (MSM) and respondent-driven sampling (persons who inject drugs and heterosexual persons) in NHBS surveys from 2008 to 2016. NHBS sampling procedures have been previously described (*10*). Persons were eligible to participate if they resided in a participating city, could complete the survey in English or Spanish, and met cycle-specific inclusion criteria (MSM: born male, aged ≥18 years, identified as male, and had oral or anal sex with another man; persons who inject drugs: aged ≥18 years, injected drugs in the past 12 months; and heterosexual persons: male or female [not transgender], aged 18–60 years, had sex with a member of the opposite sex in the past 12 months, never injected drugs, and met low income or low education criteria).^§^ For inclusion in current analyses, participants must have tested negative during the NHBS cycle, MSM must have had sex with another man in the past 12 months, and persons who inject drugs must have been male or female (not transgender). Data were analyzed by sex, age, and race/ethnicity (American Indian or Alaska Native; Asian; black or African American [blacks]; Hispanic or Latino; Native Hawaiian or Other Pacific Islander; white; and multiple race).

## Results

**FIGURE 1:**
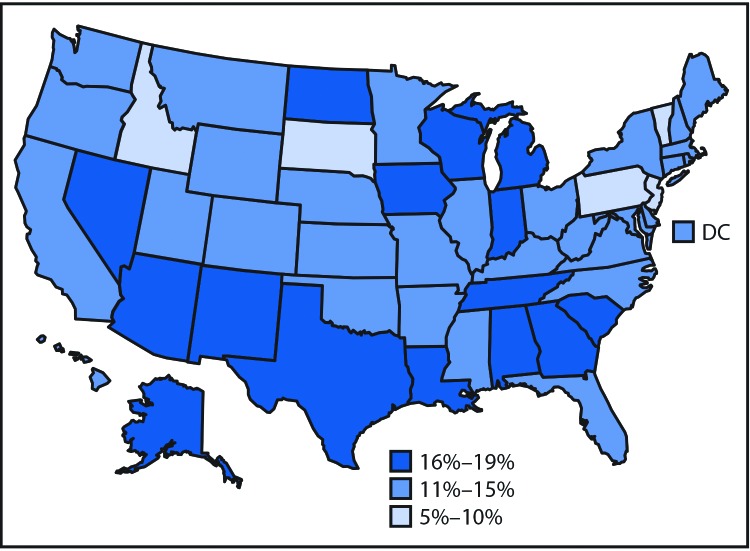
Percentage of undiagnosed infections*^,†^ among persons aged ≥13 years^§^ living with diagnosed or undiagnosed human immunodeficiency virus (HIV) infection — United States, 2015 * Overall percentage of undiagnosed infections = 14.5%. ^†^ Data classified manually. ^§^ Estimates were derived by using HIV surveillance data for persons aged ≥13 years at diagnosis in the 50 states and the District of Columbia.

In 2015, among 1,122,900 persons living with HIV infection, 162,500 (14.5%) were unaware of their infection. The percentage of undiagnosed HIV infections ranged from 5.7% to 18.5% across states (Figure 1); 50.5% of undiagnosed infections were in the South. Among 39,720 persons with HIV infection diagnosed in 2015, 21.6% had stage 3 infection (AIDS) at the time of diagnosis, and the estimated median interval from HIV infection to diagnosis was 3.0 years (Table 1). Diagnosis delays were longer among persons who were older at diagnosis than among those who were younger (median = 4.5 years among persons aged ≥55 years compared with 2.4 years among persons aged 13–24 years) (p<0.01). By race/ethnicity, median diagnosis delay ranged from 2.2 years among whites to 4.2 years among Asians (p<0.01). Diagnosis delay was longer among males (median = 3.1 years) than among females (median = 2.4 years) (p<0.01). By transmission category, diagnosis delay was longest among males with infection attributed to heterosexual contact (median = 4.9 years).

**TABLE 1:** Diagnoses of human immunodeficiency virus (HIV) infection and estimated median number of years infected at time of HIV diagnosis ― National HIV Surveillance System, United States, 2015

Characteristic	HIV diagnoses No. (%)	Years infected at time of diagnosis		
		Median	Interquartile range*	p-value^†^
**Total**	**39,720**	**3.0**	**(0.7–7.8)**	**—**
**Sex**				
Male	32,294 (81.3)	3.1	(0.7–7.8)	Referent
Female	7,426 (18.7)	2.4	(0.6–8.0)	<0.01
**Age group at diagnosis (yrs)**				
13–24	8,956 (22.5)	2.4	(0.7–5.6)	Referent
25–34	13,059 (32.9)	2.6	(0.6–7.6)	0.27
35–44	7,669 (19.3)	3.5	(0.7–9.6)	<0.01
45–54	6,306 (15.9)	4.0	(0.8–10.6)	<0.01
≥55	3,730 (9.4)	4.5	(0.8–0.6)	<0.01
**Race/Ethnicity**				
American Indian or Alaska Native	195 (0.5)	3.4	(0.7–7.7)	0.06
Asian	938 (2.4)	4.2	(0.9–8.2)	<0.01
Black or African American	17,331 (43.6)	3.3	(0.7–7.6)	<0.01
Hispanic or Latino	9,678 (24.4)	3.3	(0.7–8.2)	<0.01
Native Hawaiian or Other Pacific Islander	80 (0.2)	3.9	(0.8–8.2)	0.01
White	10,445 (26.3)	2.2	(0.6–7.6)	Referent
Multiple races	1,053 (2.7)	3.0	(0.7–8.6)	0.01
**Transmission category**				
**Male**				
Male-to-male sexual contact	26,459 (81.9)	3.0	(0.7–7.4)	<0.01
Injection drug use	1,343 (4.2)	2.9	(0.7–8.2)	<0.01
Male-to-male sexual contact and injection drug use	1,270 (3.9)	2.1	(0.6–7.3)	<0.01
Heterosexual contact	3,187 (9.9)	4.9	(0.8–11.5)	Referent
**Female**				
Injection drug use	1,004 (13.5)	2.0	(0.6–5.4)	<0.01
Heterosexual contact	6,401 (86.2)	2.5	(0.6–8.5)	<0.01

* The interquartile range describes the middle 50% of values (i.e., 25% of values are less than the lower value and 25% are greater than the higher value).

^†^ p-value is for testing the difference between two medians.

**FIGURE 2:**
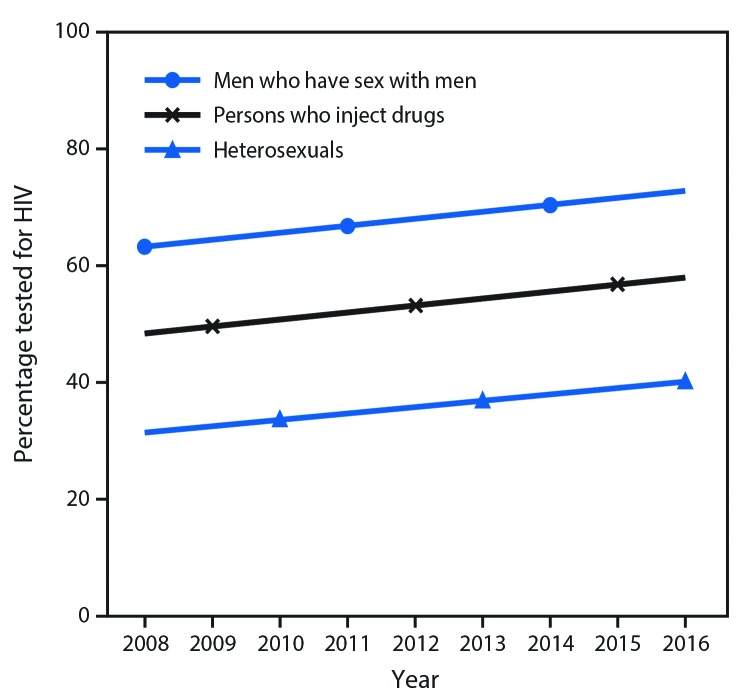
Percentage of persons tested for human immunodeficiency virus (HIV) in the past 12 months among men who have sex with men, persons who inject drugs, and heterosexual persons at increased risk for infection — National HIV Behavioral Surveillance (NHBS), United States, 2008–2016* * Data include all participants with complete valid survey data who tested negative during NHBS and cycle-specific inclusion criteria: men who have sex with men (born male, identified as male, and had oral or anal sex with another man); persons who inject drugs (injected drugs in the past 12 months); heterosexual persons at increased risk (male or female [not transgender], had sex with a member of the opposite sex in the past 12 months, never injected drugs, and met low income [not exceeding U.S. Department of Health and Human Services poverty guidelines] or low education [high school education or less] criteria). Groups are mutually exclusive.

Among persons interviewed through NHBS, the percentage reporting an HIV test in the 12 months preceding the interview increased over time among MSM (from 63% in 2008 to 71% in 2014), persons who inject drugs (from 50% in 2009 to 58% in 2015), and heterosexual persons at increased risk for infection (from 34% in 2010 to 41% in 2016) (Figure 2). The prevalence of testing in the past 12 months was higher among females than among males, among both persons who inject drugs (males, 57%; females, 59%), and heterosexual persons at increased risk (males, 39%; females, 42%). Prevalence of testing was also higher among black persons who inject drugs (and heterosexual Asians, although the numbers were small) than among persons of other race/ethnicity and persons aged 25–34 years (and persons aged 35–44 years who inject drugs) than among other age categories in each risk group (Table 2).

**TABLE 2:** Human immunodeficiency virus (HIV) testing in the past 12 months, reasons for not testing, and missed opportunities for testing among men who have sex with men, persons who inject drugs, and heterosexual persons* at increased risk for acquisition of HIV infection — National HIV Behavioral Surveillance, United States, 2014–2016

Characteristic	MSM, 2014^†^		Persons who inject drugs, 2015^§^		Heterosexual persons at increased risk, 2016^¶^	
	No. in sample	No. (%)	No. in sample	No. (%)	No. in sample	No. (%)
**HIV testing, past year (overall)**	**6,834**	**4,862 (71.1)**	**9,574**	**5,537 (57.8)**	**7,256**	**2,972 (41.0)**
**Sex**						
Male	6,834	4,862 (71.1)	6,905	3,962 (57.4)	3,257	1,276 (39.2)
Female	NA	NA	2,669	1,575 (59.0)	3,999	1,696 (42.4)
**Race/Ethnicity**						
American Indian or Alaska Native	43	31 (72.1)	94	52 (55.3)	51	19 (37.3)
Asian	144	106 (73.6)	27	15 (55.6)	11	6 (54.6)
Black or African American	1,536	1,169 (76.1)	3,098	1,948 (62.9)	5,205	2,398 (46.1)
Hispanic or Latino	1,932	1,316 (68.1)	2,145	1,214 (56.6)	1,461	368 (25.2)
Native Hawaiian or Other Pacific Islander	37	31 (83.8)	15	6 (40.0)	21	5 (23.8)
White	2,789	1,951 (70.0)	3,804	2,081 (54.7)	216	58 (26.9)
Other/Multiple races	317	230 (72.6)	374	211 (56.4)	279	114 (40.9)
**Age group (yrs)**						
18–24	1,576	1,169 (74.2)	570	312 (54.7)	1,504	628 (41.8)
25–34	2,656	2,024 (76.2)	2,317	1,379 (59.5)	1,843	870 (47.2)
35–44	1,198	836 (69.8)	2,108	1,254 (59.5)	1,401	601 (42.9)
45–54	966	588 (60.9)	2,458	1,397 (56.8)	1,727	604 (35.0)
≥55	438	245 (55.9)	2,121	1,195 (56.3)	781	269 (34.4)
**Persons not tested who visited an HCP in past year**	**1,971**	**1,325 (67.2)**	**4,036**	**2,887 (71.5)**	**4,284**	**3,205 (74.8)**
**Persons not tested who visited an HCP in the past year but were not offered an HIV test**	**1,316**	**1,052 (79.9)**	**2,849**	**2,146 (75.3)**	**3,183**	**2,511 (78.9)**
**Main reason for not testing, no. (column %)**						
Think risk for infection is low	—	896 (45.2)	—	790 (19.6)	—	740 (17.3)
Afraid of finding out they had HIV	—	327 (16.5)	—	836 (20.8)	—	642 (15.0)
Didn’t have time	—	203 (10.3)	—	467 (11.6)	—	532 (12.4)
Some other reason	—	89 (4.5)	—	195 (4.8)	—	164 (3.8)
No particular reason	—	466 (23.5)	—	1,737 (43.2)	—	2,201 (51.4)
**Total**	**—**	**1,981 (100.0)**	**—**	**4,025 (100.0)**	**—**	**4,279 (100.0)**

**Abbreviations:** HCP = health care provider; MSM = men who have sex with men; NA = not applicable.

* Aged 18–60 years.

^†^ Data include all participants with complete, valid survey data from 20 cities who reported having sex with another man in the 12 months before interview and who had negative HIV test results.

^§^ Data include all participants with complete, valid survey data from 20 cities who reported male or female gender and who had negative HIV test results.

^¶^ Data include all participants with complete, valid survey data from 17 cities who reported male or female gender, who ever had sex with a member of the opposite sex, never injected drugs, and who had negative HIV test results.

Among persons interviewed through NHBS who were not tested in the past year, most MSM reported that their main reason for not testing was that they believed their risk for infection was low, whereas most persons who inject drugs and heterosexual persons at increased risk reported that they had no particular reason for not testing. In each risk group, at least two thirds of persons who did not have an HIV test had seen a health care provider in the past year (Table 2). Among those who had not tested in the past year and had visited a health care provider, approximately three quarters reported not having been offered an HIV test at any of their health care visits.

## Discussion

Fifty percent of persons with HIV infection diagnosed in 2015 had been infected for at least 3 years, and a quarter had been infected for ≥7 years. Diagnosis delays varied substantially by population. Although the percentage of persons testing increased over time among groups at high risk, overall, 15% of persons were unaware of their infection. The prevalence of persons unaware of their infection varied among states, and half (50.5%) of persons with undiagnosed HIV infection in 2015 were living in the South. Gaps in testing remain, and missed opportunities for testing at health care visits are prevalent. Improved testing coverage and frequency are needed to meet the goal of at least 90% of persons living with HIV knowing their infection status and to reduce diagnosis delays and ultimately reduce HIV incidence in the United States (*11*).

Cultural factors (e.g., stigma, fear, discrimination, and homophobia) might contribute to longer diagnosis delays in some populations (*12*). Asians accounted for the highest percentage of persons living with undiagnosed HIV infection compared with all other race/ethnicity groups (*13*). Although blacks were more likely than whites to report testing in the past 12 months across all groups at risk, the median diagnosis delay was 1 year longer for blacks (median = 3.3 years) than for whites (median = 2.2 years). The testing results might reflect national efforts to improve access to testing among blacks, and black MSM in particular, through prevention programs and media campaigns. In 2007, CDC launched the Expanded Testing Initiative (https://www.cdc.gov/hiv/policies/eti.html) to facilitate HIV diagnosis and linkage to care among blacks and continues to support high levels of testing. CDC’s MSM Testing Initiative (https://www.researchgate.net/publication/287201580) scaled up HIV testing and linkage-to-care activities among black and Hispanic or Latino MSM in 11 cities. In addition, CDC implemented Testing Makes Us Stronger (https://www.cdc.gov/actagainstaids/campaigns/tmus), a public education campaign to increase testing among black MSM, from 2011 to 2015.

The longer diagnosis delay among non-white racial/ethnic groups might partly reflect the higher proportion of infections attributable to heterosexual contact among these groups compared with whites (*14*), given that heterosexual persons had longer diagnosis delays. Among all transmission categories, males with infection attributed to heterosexual contact had the longest median diagnosis delay (4.9 years). This observation was consistent with the finding that heterosexual males at increased risk for infection were less likely to report testing in the past 12 months than were heterosexual females at increased risk. Heterosexual men are less likely to visit a health care provider than are both women and MSM, leading to fewer opportunities for testing (*15*). Moreover, compared with other risk groups, heterosexual persons at increased risk were less likely to have been offered an HIV test even when visiting a health care provider in the past 12 months, possibly because of low perceived risk for infection (*15*,*16*). This finding highlights the importance of implementing routine screening in health care settings.

A previous estimate^¶^ of diagnosis delays among persons who received a diagnosis of HIV infection in 2011 indicated that half had been infected for 3.6 years. The median diagnosis delay of 3.0 years among HIV diagnoses in 2015 reflects an absolute reduction of 0.6 years (7 months) and a relative reduction of 17%, representing a considerable decrease over a 4-year period (*8*). Earlier detection of HIV combined with prompt linkage to care and initiation of antiretroviral treatment enhances preservation of immune function and, if viral suppression is achieved and maintained, reduces risk for sexual transmission of HIV (*4*). In addition, persons who know they have HIV infection substantially reduce their HIV-related risk behaviors: the prevalence of unprotected anal or vaginal intercourse was found to be 53% lower among persons aware of their HIV status than among those who were unaware of their status (*17*).

For HIV treatment to be effective in reducing HIV incidence, infections need to be diagnosed as quickly as possible. This requires increasing HIV testing coverage and frequency. CDC recommends testing all persons aged 13–64 years at least once as a routine part of medical care and more frequent testing (at least annually) for persons at high risk for HIV infection (*7*). A large proportion (84%) of HIV sexually transmitted from MSM and heterosexual persons is transmitted by MSM (*1*). Some sexually active MSM might benefit from more frequent testing (e.g., every 3 to 6 months) (*18*). Testing according to CDC guidelines is critical to diagnosing HIV infection, so that anyone who receives a diagnosis of HIV infection can start antiretroviral treatment. Overall, prior year testing increased among groups at high risk over time. However, 29% of MSM (in 2014), 42% of persons who inject drugs (in 2015), and 59% of heterosexual persons at increased risk (in 2016) did not report testing in the past 12 months. In addition, it is important to note that these data are from persons residing in large metropolitan statistical areas in the United States. Studies have found that persons residing in rural areas are less likely to report prior HIV testing, including in the past 12 months, compared with their urban counterparts, and that persons living in rural areas are more likely to have HIV infection diagnosed at a late stage (*19*,*20*). Barriers to implementing routine testing include lack of time, competing priorities, and concerns about reimbursement on the health care provider’s part and stigma and lack of perceived risk on the client’s part (*21*). Lack of perceived risk was also one of the main reasons cited by MSM in NHBS for not testing in the past 12 months.

A recent analysis of HIV testing frequency using NHBS data indicated that among persons at high risk for HIV infection who were ever tested, the estimated average interval between two successive HIV tests decreased from 10.5 months (2009) to 7.7 months (2014) among MSM, from 14.4 months (2009) to 11.5 months (2015) among persons who inject drugs, and from 21.1 months (2010) to 19.9 months (2013) among heterosexual persons at increased risk for HIV acquisition (*22*). Although the decreases in testing intervals are encouraging and indicate that, on average, MSM and persons who inject drugs are meeting recommendations for annual testing, these data are among persons already testing. Limited data suggest that MSM who have never been tested for HIV might engage in higher risk behaviors than do MSM who have been previously tested. One study found that MSM who had never been tested were 1.46 times as likely (95% confidence interval = 1.17–1.81) to report condomless anal sex in the past 3 months with an HIV-positive or serostatus-unknown partner than were persons who tested previously (*23*).

The findings in this report are subject to at least four limitations. First, missing CD4 test results could be caused by either incomplete reporting or not having had a CD4 test done. However, 89.4% of persons with HIV infection diagnosed in 2015 had a first CD4 test after diagnosis reported by June 2017. Second, adjustment for missing risk factors might be inaccurate if factors associated with these were not accounted for in the model. Third, NHBS is not a nationally representative sample, so results are not generalizable to all cities or to all groups at high risk in participating cities. Finally, behavioral data are self-reported and subject to social desirability bias.

The interval from HIV infection to diagnosis has decreased in recent years, but diagnosis delays continue to be substantial for some population segments. Whereas testing in the past 12 months has increased in recent years among groups at high risk, a high proportion of persons in all risk groups remain untested, with many missed opportunities for testing. Diagnosis delays lead to missed opportunities for HIV care and treatment and prolong the time a person is unaware of their infection, increasing the potential for HIV transmission. For care and treatment to reduce HIV incidence effectively, a high proportion of cases need to be diagnosed and treated soon after infection occurs. Continued efforts to determine why cases are not being diagnosed soon after infection and to assure implementation of routine and targeted testing can help reduce both the number of persons unaware of their infection and diagnosis delays.

**Key Points**

• Persons unaware of their human immunodeficiency virus (HIV) infection account for approximately 40% of ongoing transmissions in the United States.

• Eighty-four percent of sexual transmission from men who have sex with men (MSM) and heterosexuals is estimated to occur from MSM.

• Among persons with HIV infection diagnosed in 2015, the estimated median interval from infection to diagnosis was 3 years.

• Prior year testing increased over time among groups at high risk for HIV infection. However, 29% of MSM, 42% of persons who inject drugs, and 59% of heterosexual persons at increased risk did not report testing in the past 12 months.

• In each risk group, at least two thirds of persons who did not have an HIV test had seen a health care provider in the past year.

• Continued efforts to ensure routine and targeted testing can help reduce the number of persons who are unaware of their infection, diagnosis delays, missed opportunities for care and treatment, and HIV transmission.

• Additional information is available at https://www.cdc.gov/vitalsigns/.

## Footnotes

* Past year testing was assessed during the interview by asking participants, “When did you have your most recent HIV test? Please tell me the month and year.” A missed opportunity was defined as a visit to a health care provider in the past 12 months for a person who did not report past year HIV testing or as not being offered an HIV test at any health care visits for a person who did not report past year HIV testing and had visited a health care provider in the past year.

§ Social-structural variables were used to identify a representative sample for NHBS of heterosexual persons at increased risk of HIV infection. Heterosexual persons at increased risk were defined as male or female (not transgender) in a metropolitan statistical area with high AIDS prevalence, who had sex with a member of the opposite sex in the past 12 months, never injected drugs, and met low income or low education criteria. Low income was defined as not exceeding U.S. Department of Health and Human Services poverty guidelines and low education as having a high school education or less.
